# Distilled vision transformers with CNN fusion for robust cashew apple maturity prediction

**DOI:** 10.3389/fpls.2026.1787609

**Published:** 2026-04-21

**Authors:** Sumalatha Lingamgunta, Jeevaratnam Mudidana, Durai Raj Vincent, Felicita S. A. M.

**Affiliations:** 1 Department of Computer Science and Engineering, University College of Engineering Kakinada, Jawaharlal Nehru Technological University Kakinada, Kakinada, Andhra Pradesh, India; 2 School of Computer Science Engineering and Information Systems, Vellore Institute of Technology, Vellore, India

**Keywords:** cashew apple, ConvNeXt, EdgeNeXt, knowledge distillation, maturity grading, vision transformer

## Abstract

**Introduction:**

Cashew apple is a nutrient-rich fruit containing abundant minerals, vitamins, and energy. However, its fleshy texture and delicate skin significantly limit its storage life and market value. Accurate maturity grading is therefore essential for improving post-harvest management and transportation efficiency.

**Methods:**

This study proposes a lightweight vision transformer (ViT) student model trained using multi-granular knowledge distillation (KD) from a stronger data-efficient image transformer (DeiT)-Base teacher. The distillation framework integrates response-based soft-label supervision, attention transfer, and token-level feature regression to enhance representation learning under limited data conditions. Auxiliary lightweight architectures, including MobileNet, ConvNeXt, and EdgeNeXt, were trained independently to provide complementary predictions, and a weighted fusion strategy was employed for ensemble evaluation.

**Results:**

The proposed ensemble ViT-KD with EdgeNeXt achieved 90% accuracy under the evaluated test split. To ensure statistical reliability and address potential partition bias, a stratified fivefold cross-validation was conducted on the dataset, yielding a mean accuracy of 86.89% ± 2.89% with consistent F1 scores and recall. The relatively low variance across the folds indicates stable internal generalization. Comparative experiments with conventional convolutional neural network (CNN) baselines and lightweight CNN baselines such as MobileViT-S and ShuffleNetV2 were performed, with the proposed ensemble framework achieving improved accuracy while maintaining computational efficiency. Computational analysis indicates that the stand-alone distilled ViT maintains a real-time inference capability of 8.79 ms per image, which supports suitability for edge-oriented agricultural applications.

**Discussion:**

These results highlight the effectiveness of knowledge-distilled lightweight transformers for data-efficient maturity grading of cashew apples.

## 1 Introduction

Cashew apple has been brought to India by the Portuguese in the 16th century. It is fleshy and holds a cashew nut at the bottom. The maturation and ripening of cashew apples go through phases including green to soft and juicy, with the outer skin color changing from red, orange, and yellow. Cashew apple is nutrient-rich and packed with minerals and vitamins, hence has many nutritional and medicinal uses. However, certain factors such as the formation of an abscission layer, the apple size increase, thin skin, and the low shelf storage at the ripened state limit its large-scale utilization, transportation, and commercialization.

Determining the maturity of cashew apples is important in ascertaining the quality of cashew nuts and juice production. Accurate grading of cashew apples based on their maturity level ensures better processing, reduced wastage, and higher commercial value (Aherwadi et al., 2022). However, manual grading is time-consuming and may sometimes miss certain details. Moreover, it is challenging under varying lighting conditions and with diverse fruit appearances in real farm environments (Gupta et al., 2025).

Cashew apples have received very little attention despite the effective application of deep learning for ripeness identification, although major fruits such as bananas, mangoes, and apples have already been extensively used. Maturity classification is a more difficult and understudied subject as the visual and textural characteristics of cashew apples differ greatly from those of traditional fruits (Das and Arora, 2017; Colaco and Kamat, 2025).

Not much work has been carried out with fruit maturity grading, which is a binary problem, whereas cashew apples have three distinct stages. These three classes are the premature stage, the mature stage, and the over-ripened stage. These stages are basically differentiated by the color tone and the surface structure of cashew apple. The not much visible differences between these stages pose difficulty in correctly classifying this model (Winklmair et al., 2025).

Data collection is a difficult task in the selected domain as the unavailability of quality data is a current issue. Many of the available datasets were taken in controlled environments with clean backgrounds and good lighting. Such conditions restrict generalization of the model as images taken in a normal environment have background clutter and shadow effects. Furthermore, there is a conspicuous dearth of study on the maturity assessment of cashew apple, mostly concentrating on kernel quality, size, and flaws. Specialized models are required to accurately capture these differences as the surface features and color transitions of the apple play a significant role in determining harvest readiness.

The maturity grading of cashew apples is vital because it affects the taste, yield, and nut processing. Images taken in the field have illumination issues, peculiarities, and background. The datasets are small, with fewer images in the majority of cases. To analyze the ripeness for maturity grading, the use of transformers is challenging as standard vision transformers (ViTs) are heavy and data-hungry and use long-range color or shape features. In this work, a model is proposed where small ViT, which is token-efficient, with knowledge distillation (KD) was used to transfer inductive bias from a larger teacher.

ViT, which was introduced in 2020, follows the same transformer architecture, originally developed for natural language processing. A major shift in the image perception and interpretation is influenced by ViTs. Traditional convolutional neural networks (CNNs) use spatially local filters to capture patterns. In contrast, ViTs consider an image as a sequence of patches that learn to understand the relationships among these patches using self-attention mechanisms (Dosovitskiy et al., 2020; Siricharoen et al., 2023; Zhang and Zhang, 2025).

ViTs transform images into feature vectors, forming sequences similar to words in sentences by dividing the input image; for instance, 224 × 224 pixels is divided into 32 × 32 patches, and each patch is flattened for projecting it linearly into a feature vector. These feature vectors, called tokens, are then processed through an encoder that uses multi-head self-attention to model global dependencies among patches.

With this transformation, the ViT looks at all parts of the image simultaneously and learns how one region relates to another, such as the relation between the top color and the texture at the bottom, or how the combination of color and shape indicates ripeness. This quality of ViTs makes them different from CNNs. ViTs leverage self-attention to capture long-range dependencies across the image patches. Models such as ViT-B16 are often too large and resource-intensive for many practical applications. Such large models, although powerful, are slow and unsuitable for real-time deployment with limited computational capacity.

Maturity grading of cashew apples is challenging in this work due to the chosen data being small. For real-time operation at farms, resource-intensive models are constrained. Lightweight models are efficient and generalize from limited data without overfitting. During the literature survey, we did not find many papers that address the cashew apple-based maturity grading problem, which is the rationale for this work. The selected dataset is also smaller, which additionally poses a significant challenge (Alves de Moraes et al., 2024; Charukeshi et al., 2024; Wakchaure et al., 2024; Feng et al., 2025; Hassija et al., 2025).

By considering these limitations, the model proposed here combines tiny ViT as the KD setup, where the model retains the global attention mechanism of ViT while having the capability of the technique to handle this small data with considerably reduced computational costs (Palma et al., 2019). This work involves the cashew apple maturity grading task, where the ViT-based distillation model is proposed to solve this small problem with limited data and limited variation among data. The key contributions of this work are outlined below.

1. From the extensive literature review, we found that very few studies have considered cashew apple for ripening prediction, making this work relatively underexplored, but important.
2. This work takes into consideration a small dataset of Goa cashew apple images, which makes classification challenging due to the extremely limited data and not much inter-class variations. To address this problem, three different models were experimented on and evaluated for the selection of the best-performing model.
3. The first model utilizes the baseline distillation framework by integrating MobileNetV3-Small as an auxiliary learner in a hybrid ViT–CNN ensemble framework.
4. The second model is considered with advanced hybrid architecture by combining the student ViT and ConvNeXt-Tiny, enhancing its capable CNN-based feature extraction to form a robust framework capable of delivering good cashew maturity detection.
5. The third model combines KD from a DeiT-Base teacher to a ViT-Tiny student, with EdgeNeXt-Small trained in parallel to provide proper feature support.
6. The experimental results indicate that the models proposed in this work outperformed traditional CNN-based approaches, with one of the three proposed frameworks achieving the best results.

The remainder of the paper is organized into literature survey, proposed model and evaluation of the results, and comparative analysis, followed by the conclusion.

## 2 Literature survey

From the literature, several similar tasks were performed with a CNN-based architecture, which produced very good results; however, it suffers in a few cases, such as in fruit maturity grading tasks. These models struggle to generalize well on small agricultural datasets, which are common in real-world farm scenarios. ViTs have shown remarkable success in image understanding, but their high computational costs and data dependency make them unsuitable for small datasets and low-resource deployment environments, such as handheld devices or Internet of things (IoT)-based monitoring systems.

In Goyal et al (Goyal et al., 2024), an ensemble method with various regressors, including support vector machine (SVM), decision trees, random forest, and gradient-based method, was utilized for the task. The different characteristics of tomato were used for determining quality. Various attributes such as the size, color, shape, texture, taste, nutritional content, defects, and ripeness were considered, and 2,048 automated features were generated in this model. Principal component analysis (PCA) dimensionality reduction was applied, and automated features of a final selection of 50 features were generated. The proposed method used a stacking technique to predict the shelf life of tomato with an accuracy rate of 90.35%.

The survey presented in Wang et al (Wang et al., 2022) outlined the techniques used for the classification and grading of fruits proposed in various research works. Another work (Tapia-Mendez et al., 2023) used a classification system with CNN to determine fruit ripening. The system was trained and tested on three fruit varieties, i.e., mango, apple, and banana, and limits itself to only these three varieties. In Papandreou et al (Papandreou et al., 2015), a CNN-based model used for semantic segmentation of an image at pixel level to classify objects was proposed. Some works proposed deep learning methods for segmentation to detect edges in the color images of berries to identify different growth patterns and characteristics.

Another study (Zabawa et al., 2019) considered six deep learning models, i.e., YOLOv3, YOLOv3-SPP, YOLOv3-Tiny, YOLOv4, YOLOv4-Small, and YOLOv4-Tiny, for detecting ripeness in wild blueberries. These networks focused on three types (green berries, red berries, and blue berries) and two-class (unripe and ripe berries) models. One of the models used 1,280 × 736 images and achieved the highest F1 score of 0.82.

The deep convolutional neural network (DCNN) for fruit ripeness identification proposed in Grimm et al (Grimm et al., 2019) uses volatile organic compounds (VOCs) from fruit samples collected from metal oxide semiconductor (MQ) gas sensors. The method in Craig et al (MacEachern et al., 2023) feeds images taken at different ripeness degrees to an artificial intelligence (AI)-based fruit maturity identification system to output fruit ripeness.

Another work (Hasan et al., 2022) used CNN for cashew fruit identification from images with better accuracy. In MacAtangay et al (MacAtangay et al., 2022), a maturity assessment device was proposed using Raspberry Pi to classify custard apple into five maturity stages: 0%, 25%, 50%, 75%, and 100% areole opening. The images were pre-processed, segmented, and analyzed to extract relative red–green–blue (RGB) color components, which were correlated with chemical properties such as total soluble solids (TSS). *K*-means clustering and SVM classifiers were implemented on the Raspberry Pi using Python for on-device image classification. The statistical analysis showed clear difference in the R, G, and TSS values across various maturity stages, resulting in better grading accuracy. As the device is inexpensive and potable, it is ideal for farm use and can be adapted to other fruits with minimal changes to the algorithm, providing a practical low-cost alternative to commercial grading systems.

In another study, Anticuando et al (Anticuando et al., 2022) introduced an integrated explainable artificial intelligence (XAI) for the classification of carambolas across different maturity levels. It evaluates both ResNet and ViT models and boosts their interpretability using Grad-CAM, attention maps, and random forest to highlight the most influential image regions and features. The proposed XAI-enabled approach provided clear visual and feature-level explanations of the classification outcomes, achieved better accuracies with ResNet and ViT, and demonstrated strong potential for standardized and rapid maturity assessment of carambolas and other fruits.

Another work (Feng et al., 2025) introduced a comprehensive framework for strawberry maturity classification by combining deep learning techniques with chemical and spectral analyses. Three deep learning models—RegNet_Y_800MF, RegNet_Y_8GF, and Swin Transformer V2 Tiny—were developed and evaluated. Among the tested models, the Swin-based model achieved better accuracy on the test set, surpassing other variants. Other research works considered for the literature are provided in **Table 1**.

**Table 1:** Summary of works proposed on fruit ripening or maturity grading.

Year	Reference	Fruit	Task	Model	Dataset	Result/report
2024	Wang et al., 2024	Tomato	Ripeness detection in complex scenes	Improved YOLOv8 (Swin Transformer+DSConv+Focal-EloU)	Tomato dataset	mAP50 ≈ 86.9% Recall: 82.0%, ~190 FPS
2025	Martínez-Mora et al., 2025	Banana	Ripeness and quality attributes	CNN and VGG variants	1565 RGB images across 20 day cycle	CNN = 90.4% accuracy VGG = 89.2%
2025	Han and Yi, 2025	Palm oil fruit	Five-level maturity classification	ResNet50 and InceptionV3(TL) *vs*. Shallow CNN	More than 8000 images	Test accuracy >85% (ResNet50, ~86.4%) Discussion on generalization
2023	Tapia-Mendez et al., 2023	Multi-fruit	Fruit type + ripeness (fresh/rotten)	Two MobileNetV2 models (TL)	3,360 images (32 classes total; 6 with ripeness)	Fruit/vegetable classification: 97.86% accuracy; ripeness: 100% accuracy (dataset-specific)
2025	Chuquimarca et al., 2025	Banana	Robustness under lighting shifts	ViT, Inception V3 with LIME/Gamma augmentation	Real banana Images with multiple illuminations	Baseline, ~92.5% (ideal light); drops under gamma; augmentation restores ~+8.3 pp
2024	Xiao et al., 2024	Apple	Ripeness identification	Transformer/YOLO	Variety of apple images	Reported maturity classification accuracy ranged from 90% to 97%

DL, Deep Learning; TL, Transfer Learning; RGB, Red–Green–Blue; mAP, Mean Average Precision; FPS, Frames Per Second. DL, Deep Learning; TL, Transfer Learning; mAP, Mean Average Precision; FPS, Frames Per Second.

The main technique used in this work, knowledge distillation (KD), was proposed for the purpose of compression, where the student model learns from the large teacher model. The soft probability distributions provide better supervisory signals, unlike the hard labels that provide improved generalizations. This basically provides smoother decision boundaries especially when the data size is extremely limited (Alkhulaifi et al., 2021).

The concept of KD extended to ViT has been impacted in the development of data-efficient image transformers (DeiTs). This enables transformers with high data requirements to effectively handle limited datasets using distillation-based supervision. Long-range dependencies are a powerful tool through self-attention mechanisms, which are basically data-hungry and overfit when the model is not sufficiently trained with adequate data. Distillation enables the transfer of semantic structure and inductive bias from a high-capacity teacher model to a lightweight student architecture, improving stability and predictive consistency (Touvron et al., 2021).

When the focus of deployment arises, lightweight models such as EdgeNeXt or MobileNetV3 are proposed, which use depth-wise separable convolutions, channel attention modules, and parameter-efficient blocks (Maaz et al., 2022). This type of efficient and lightweight architecture is helpful in environments such as agriculture farmlands, where real-time deployments are required in a remote scenario. Few-shot learning is also a relevant option in precision agriculture, whereas crop-specific geographically constrained data will pose a different challenge (Kamilaris and Prenafeta-Boldú, 2018). A structured transfer learning approach can effectively reduce the variance in limited sample scenarios. In such cases, hybrid CNN–transformer architectures with KD provide a practical method to enhancing robustness without relying on large datasets.

With the above literature review and how we derived the proposed architecture, the need for fruit maturity grading using deep learning-based algorithms is clearly established, particularly for the timely classification of appropriate maturity levels. An extensive review of existing studies revealed that very limited work has been carried out on cashew apple-based maturity analysis, making it a relatively underexplored yet important research gap. Such a system can improve harvest timing and post-harvest handling while reducing wastage and enhancing value addition in the cashew industry. The following sections present the proposed methods along with their experimental evaluation.

## 3 Proposed methods

### 3.1 Dataset

The proposed framework categorizes cashew apples into three maturity levels: unripe, ripe, and overripe. Every image that is processed is resized to a standard 224 × 224 pixel resolution so that it fits perfectly into the ViT and ConvNeXt models. These models are trained on the cashew dataset, which was downloaded from IEEE Dataport (Sawant, 2025), with a total size of approximately 50.7 MB and contains 900 image samples. These samples are evenly distributed across the three maturity classes, with 300 images per class, resulting in a balanced dataset that supports fair class-wise evaluation.

**Figure 1:**
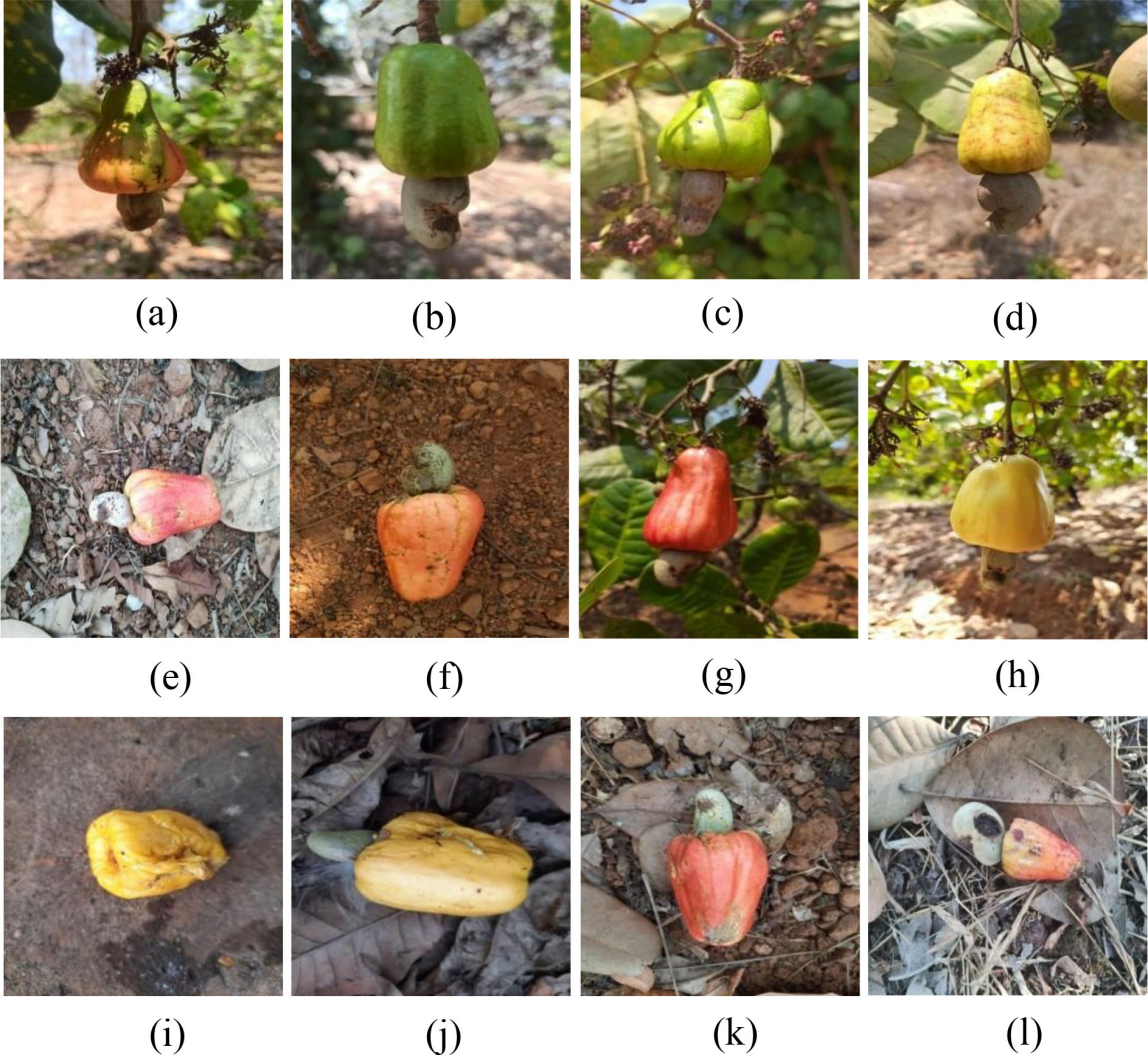
The three classes in the dataset. **(A–D)** Unripe. **(E–H)** Ripe. **(I–L)** Overripe.

The main constraint of the selected dataset is that the overall size is only 900 images put together, which is the main drawback across much of precision agricultural research. There is often unavailability of large-scale annotated datasets. With the available dataset, high-capacity transformer architectures may overfit. For mitigating the constraint only, the proposed framework was chosen in such a way that it integrates both KD, extensive augmentation, and hybrid CNN–transformer fusion to enhance its robustness for the unseen samples. These design considerations were incorporated to balance model generalization with computational efficiency. Sample images representing the three classes in the dataset are shown in **Figure 1**.

This study adopts a stratified training, validation, and test splits to maintain class imbalance and ensure experimental consistency, with 70% of the dataset allocated to training, 15% to validation, and 15% to testing. Training was performed using mini-batches of 16 images per iteration to enable stable learning while also maintaining computational efficiency.

### 3.2 Vision transformer

A ViT is a deep learning architecture that applies the transformer model, which treats an image as a sequence of patches (e.g., 16 × 16), flattens them, and feeds them to a transformer encoder. It uses self-attention to understand relationships across the entire image rather than sliding convolutional filters as in a CNN. It is a powerful model, but requires large datasets. Traditional ViTs typically need large training datasets to perform well, often millions of images, without which they struggle to generalize. However, this issue can be resolved with a DeiT.

A DeiT is an improved version of ViT in which transformers are trainable on smaller datasets. DeiT uses the same patch-based transformer architecture as ViT. In addition, a KD token is added along with a classification (CLS) token. DeiT achieves competitive accuracy with less training data and is lightweight and efficient on mid-sized datasets, such as the cashew dataset. In the proposed work, the teacher network is a DeiT-Base model (deit_base_patch16_224), while the student network used is a lightweight DeiT-Tiny model (deit_tiny_patch16_224).

### 3.3 Knowledge distillation

In the proposed work, three KD-driven ViT frameworks with the parameters displayed in **Table 2** were proposed to improve the accuracy and efficiency of cashew maturity classification. All three models share a common backbone, where a powerful DeiT-Base network functions as the teacher and a lightweight DeiT-Tiny network acts as the student.

**Table 2:** Vision transformer (ViT) knowledge distillation parameters.

Parameter	Details
Teacher model	Deit_base_patch16_224
Teacher parameters (approx.)	86 million
Teacher feature dimension (CLS token)	768
Teacher training status	Frozen (requires_grad = False)
Student model	deit_tiny_patch16_224
Student parameters (approx.)	~5.7 million
Student feature dimension (CLS token)	192
Feature projector	nn.Linear(192 → 768)
KD type	Logit distillation (KL with temperature) with feature distillation (MSE) and hard-label CE
Output classes	3 (Unripe, Ripe, Overripe)
Input image size	224 × 224
Batch size	16
Transforms (Train)	1.RandomResizedCrop(224, scale=(0.7,1.0)) 2.RandomHorizontalFlip() 3.ColorJitter(0.3,0.3,0.3,0.1) 4.RandomRotation(12) 5.ToTensor() 6.RandomErasing(p=0.2) 7.Normalize([0.485,0.456,0.406], [0.229,0.224,0.225])
Transforms (Val/Test)	1.Resize(256) (224×1.14) 2.CenterCrop(224) 3.ToTensor() 4.Normalize([0.485,0.456,0.406], [0.229,0.224,0.225])
Loss functions used	CrossEntropyLoss, KLDivLoss (KD with T = 4, soft targets), MSELoss (feature distillation)

*CLS*, classification; *KD*, knowledge distillation; *MSE*, mean squared error; *CE*, cross-entropy; *KL*, Kullback–Leibler.

KD is a training technique where large, well-trained models called a “teacher” transfer its knowledge to a smaller, faster model called a “student.” The teacher learns powerful and meaningful patterns from the data, and the student is made to learn from the teacher. Normally, the student learns from dataset labels such as Unripe, Ripe, or Overripe. However, the teacher provides the knowledge about the soft probabilities of these classes, such as 70% unripe, 25% ripe, and 5% overripe. These soft outputs contain extra information about the relationships of the classes that the hard labels cannot express. The student learns from soft signals through logit distillation.

The KD lightweight student model learns from ground-truth labels and also from the predictions and feature representations of the teacher model. The overall distillation loss combines the three components: cross-entropy loss, soft-logit distillation loss, and feature-level distillation loss.

#### 3.3.1 Cross-entropy loss (hard labels)

This loss ensures that the student learns the correct class levels. The cross-entropy loss is defined in Equation 1.

(1) $$L_{\text{CE}}=-\sum_{c=1}^{C}y_{c}\text{log}({\hat{y}}_{c})$$

where $y_{c}$ is one-hot ground truth and ${\hat{y}}_{c}$ is the student-predicted probability for class *c*.

#### 3.3.2 Logit distillation (soft targets from teacher)

The teacher produces soft probabilities using a temperature, *T*.

(2) $$p_{\text{t}}(c)=\text{softmax}(\frac{z_{\text{t}}}{T})$$

The student produces.

(3) $$p_{\text{s}}(c)=\text{softmax}(\frac{z_{\text{s}}}{T})$$

The distillation uses Kullback–Leibler (KL) divergence.

(4) $$L_{\text{KLD}}=T^{2}\cdot \text{KL}(p_{\text{t}}∥p_{\text{s}})$$

Factor $T^{2}$preserves the gradient scale during training. The soft probabilities are computed as shown in Equations 2, 3, and the distillation loss is defined in Equation 4.

#### 3.3.3 Feature distillation for CLS token alignment

The student learns the teacher’s internal representations from the CLS token, i.e., the teacher’s interpretation of the image. This type of distillation is called feature distillation (FD). The CLS token is a learnable vector added to the ViT, which acts as a placeholder that collects information from all image patches through the self-attention mechanism. FD helps the student mimic the teacher’s internal representation with the help of CLS token alignment. The CLS token feature of the teacher, $f_{\text{t}}$, and the CLS token feature of the student, $f_{\text{s}}$, are compared using mean squared error (MSE). The feature distillation loss is computed as shown in Equation 5.

(5) $$L_{\text{FD}}=∥f_{\text{t}}-f_{\text{s}}{∥}^{2}$$

The overall loss function is defined in Equation 6.

(6) $$L_{\text{total}}=\alpha L_{\text{CE}}+\;\beta L_{\text{KLD}}+\gamma L_{\text{FD}}$$

where *α*, *β*, and *γ* are the weighted contributions of cross-entropy, logit distillation, and feature MSE, respectively. The distillations make the student smaller, faster, efficient, and nearly as accurate as the teacher.

The loss weighting coefficients α, β, and γ control the relative contributions of hard-label supervision, logit-based distillation, and feature-level distillation, respectively. In this study, the values α=0.6, β=0.3, and γ=0.1 were selected based on preliminary empirical tuning to balance the classification accuracy and the knowledge transfer effectiveness. Several candidate configurations were evaluated during pilot experiments, and the selected combination produced stable convergence and the best validation performance.

In this work, the first model serves as a baseline KD approach, relying solely on the interaction between the teacher and the student using a combination of cross-entropy, KL divergence, and feature-level MSE losses. Building upon this, the model integrates MobileNetV3-Small as an auxiliary learner, introducing an ensemble-style mechanism where the features from a lightweight CNN complement the representations learned by the student ViT.

The second model expands on this approach by incorporating ConvNeXt-Tiny to build a hybrid ViT–CNN ensemble that increases structural and feature diversity. In all three frameworks developed in this work, the feature alignment is the same, which allows the student model to learn from the teacher’s predictions, as well as learn from the deeper semantic information captured in the CLS token embedding. The third framework uses EdgeNeXt-Small as an auxiliary learner by moving from the ViT-KD design to hybrid ViT–CNN architectures. This framework optimizes the trade-off between computational efficiency and classification accuracy.

### 3.4 MobileNetV3-Small

MobileNetV3-Small is the lightweight version of the MobileNetV3 architecture designed for resource-constrained devices such as IoT sensors and embedded systems. Closed to only 2.5 million parameters, MobileNetV3-Small is considerably a low-parameter CNN among other architectures. This is built with inverted residual blocks along with depth-wise separable convolutions and squeeze-and-excitation attention. It enables effective feature extraction with minimal computation overhead. It uses squeeze-and-excitation attention blocks into selected layers to adaptively recalibrate channel-wise features, enabling the network to focus on the most relevant visual information. Instead of a rectified linear unit (ReLU), it uses a Hard-Swish activation function, which improves the nonlinear representation capability without adding computational overhead.

In the context of cashew apple maturity classification, MobileNetV3-Small serves as a computationally efficient baseline model. It extracts local texture and color cues that are critical for distinguishing maturity stages. The parameters of MobileNetV3-Small are provided in **Table 3**.

**Table 3:** MobileNetV3-Small parameters.

Parameter	Details
CNN model	MobileNetV3-Small
No. of parameters (approx.)	~2.5 million
Model type	Lightweight convolutional neural network with inverted residual blocks and squeeze-and-excitation (SE) attention
Training type	Standard supervised learning
Output classes	3 (Unripe, Ripe, Overripe)
Input image size	224 × 224
Batch size	16
Epochs	30
Loss function used	CrossEntropyLoss
Optimizer	AdamW
Learning rate	2 × 10^−4^
Activation function	Hard-Swish
Attention mechanism	Squeeze-and-excitation (SE) blocks
Output function	Softmax (applied during inference)

*CNN*, convolutional neural network.

### 3.5 ConvNeXt-Tiny

ConvNext-Tiny is a new generation of CNN designed to integrate the strengths of both classical CNNs and transformer-based architectures. It is designed with a set of next-level improvements inspired from ViTs, such as large-kernel depth-wise convolutions, simplified block structures, enhanced normalization methods, and simplified block designs. ConvNeXt-Tiny is a model from the ConvNext family. It has approximately 28 million parameters that offer a good trade-off between model capacity and computational efficiency, making it ideal for resource-constrained or real-time applications.

The base architecture of ConvNeXt is similar to the hierarchical multistage structure of ResNet or EfficientNet. The input image is processed across four stages, with each stage built using ConvNeXt blocks that include depth-wise convolutions, point-wise convolutions, Gaussian error linear unit (GELU) activations, and LayerNorm components. The initial layers focus on fine, local textures such as edges, colors, and shapes. The later layer focuses on larger and global patterns such as the fruit structure and the maturity patterns.

A depth-wise convolution such as a 7 × 7 kernel is applied on one filter per channel, unlike a regular convolution. This reduces the number of parameters and gives a wider view of the image. This mimics the behavior of transformers. After depth-wise convolution, a 1 × 1 convolution mixes information across all channels similar to the multilayer perceptron (MLP) layers in the ViT.

The activation function used in ConvNeXt is GELU activation, which is smoother and more expressive than ReLU, enabling the model to learn complex patterns. Subsequently, layer normalization is used to allow the model to extract rich spatial and semantic features. ConvNeXt uses an MLP structure similar to ViTs, which expands channels and shrinks back to the original size, enabling it to learn diverse patterns. For cashew apple maturity grading, ConvNeXt-Tiny complements ViTs. It is particularly effective for local texture cues such as skin patterns, surface coloration, and small maturity-related details. The parameters are displayed in **Table 4**.

**Table 4:** ConvNeXt-Tiny parameters.

Parameter	Details
CNN model	ConvNeXt-Tiny
No. of parameters (approx.)	~28 million
Model type	Modern convolutional neural network with depth-wise separable convolutions and transformer-inspired design
Training type	Standard supervised learning
Output classes	3 (Unripe, Ripe, Overripe)
Input image size	224 × 224
Batch size	16
Epochs	30
Loss function used	CrossEntropyLoss
Optimizer	AdamW
Learning rate	2 × 10^−4^
Activation function	GELU
Normalization layer	Layer normalization
Output function	Softmax (applied during inference)

*CNN*, convolutional neural network; *GELU*, Gaussian error linear unit.

### 3.6 EdgeNeXt-Small

EdgeNeXt-Small is used as a complementary convolutional branch to enhance local feature representation. It belongs to the family of CNNs augmented with Conv-Attention blocks, which enable efficient global context aggregation while preserving strong spatial inductive biases. The network operates on RGB images of size 224 × 224 × 3 and is organized into four stages, preceded by a convolutional stem with 24 channels for initial low-level feature extraction. The successive stages progressively increase the channel dimensionality from 48 to 96, 160, and 304, allowing the model to capture fine-grained textures, edge patterns, and higher-level semantic cues at multiple scales. Following global average pooling, a fully connected classification head maps the final 304-dimensional feature vectors to the target three output classes corresponding to the Unripe, Ripe, and Overripe categories. The parameters are displayed in **Table 5**.

**Table 5:** EdgeNeXt-Small parameters.

Parameter	Details
CNN model	edgenext_small
No. of parameters (approx.)	~5.6 million
Model type	Convolutional neural network with Conv-Attention blocks
Training type	Standard supervised learning
Output classes	3 (Unripe, Ripe, Overripe)
Input image size	224 × 224
Batch size	16
Epochs	30
Loss function used	CrossEntropyLoss
Optimizer	AdamW
Learning rate	2 × 10^−4^
Activation function	GELU
Output function	Softmax

*CNN*, convolutional neural network; *GELU*, Gaussian error linear unit.

### 3.7 Data augmentation

**Figure 2:**
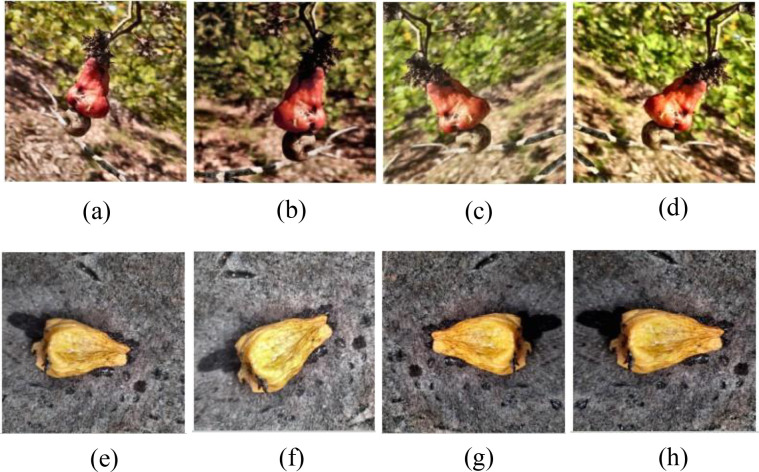
Augmented sample classes. **(A–D)** Ripe. **(E–H)** Overripe.

In the training phase, the images are applied to a set of augmentations. At first, the image is randomly cropped and resized, enabling the model to view the image at varying scales. A horizontal flip is applied later, allowing the model to learn that the fruit can appear in either direction. The ColorJitter operation adjusts the brightness, contrast, saturation, and hue, allowing the model to handle images captured under different lighting conditions. The image is rotated by a small angle to ensure that the model understands that the images are not just perfectly upright. After all these transformations, the image is converted into a tensor format and Random Erasing covers a small portion of the image, prompting the model to focus on the overall shape instead of a single region. Finally, all images are normalized to match the ImageNet statistics, ensuring stable and consistent training. A number of augmented images are shown in **Figure 2**.

For the validation and test phases, the preprocessing steps included resizing, center cropping, conversion to tensors, and normalization. This minimal pre-processing avoids any randomness that might influence the results, ensuring the model’s accuracy.

### 3.8 Model I: ViT-KD with MobileNet V3

In model I, the teacher model, DeiT-Base (deit_base_patch16_224), and the student model, DeiT-Tiny (deit_tiny_patch16_224), belong to the ViT (ViT/DeiT) family and serve as a high-capacity mentor and a lighter and more efficient variant, respectively, in the KD setup.

In the KD setup, two ViT models work together: a large, powerful teacher model and a smaller, more efficient student model. The goal is to transfer the teacher’s understanding to the student so that it can achieve strong accuracy with far fewer parameters.

The first ViT is a DeiT-Base model (deit_base_patch16_224), which was used as the teacher network. This is a powerful ViT containing approximately 86 million parameters, which allows capturing rich semantic information from images. The teacher produces a 768-dimensional CLS token embedding, which serves as the global representation of the input image. In the entire training process, the teacher model weights were not updated to enable the student model to learn from a stable and consistent reference without influencing the teacher’s behavior. The teacher produces two key outputs: soft-class probabilities called teacher logits and CLS token embedding, which serves as a compact representation of the entire image.

The student model in the framework is a lightweight DeiT-Tiny (deit_tiny_patch16_224). The student model has approximately 5–7 million parameters and generates a 192-dimensional CLS token embedding, unlike the 768-dimensional teacher’s embedding. These embeddings are aligned with a projection layer, nn.Linear(192 → 768). This layer maps the student’s CLS token into the same feature space as that of the teacher, enabling direct feature-level comparison and distillation.

During the training, the student outputs both its own logits and its projected CLS token features. It learns the correct class labels using cross-entropy loss, while the softened teacher logits are used to mimic the teacher’s decision patterns. The CLS token embeddings enable the student to imitate the teacher’s deeper semantic understanding.

The student is trained effectively using three types of losses: cross-entropy loss, KL divergence-based KD loss, and feature-level MSE loss. The cross-entropy loss teaches the student to correctly classify the input images. KL divergence-based KD encourages the student to mimic the teacher’s prediction distribution using a temperature of 4 to soften the probabilities. The feature-level MSE loss aligns the student’s CLS token features with those of the teacher’s, enabling the student to learn deeper semantic patters beyond the final outputs.

These losses are blended using simple weights: *α* = 0.6 for the cross-entropy loss, *β* = 0.3 for KL divergence, and *γ* = 0.1 for feature-level MSE. The entire student model is optimized using AdamW optimizer with a learning rate of 3*e*−4 and a weight decay of 1*e*−4. The training runs for 30 epochs using batches of 32 images resized to 224 × 224. The model details are introduced in **Table 2**.

In addition to the transformer-based models, the framework of model I also incorporates a MobileNetV3-Small (mobilenetv3_small_100) network. This model represents the lightweight convolutional branch and is optimized for efficiency through depth-wise separable convolutions, inverted residual blocks, squeeze-and-excitation mechanisms, and Hard-Swish activations. MobileNetV3 generates class logits through its final classification head and is trained independently using only the cross-entropy loss, without any distillation from the teacher. Owing to its compact design and low computational footprint, it serves as a useful complementary model in the ensemble evaluation.

Finally, a FD loss is included to align the internal representations of the student with those of the teacher. This is done using an MSE loss between the student’s projected CLS token features and the teacher’s CLS features. While the logit-based KD teaches what the teacher predicts, this feature-level alignment teaches how the teacher internally represents visual patterns. All three components are combined into a single training objective using weighted contributions: *α* = 0.6 for the cross-entropy loss, *β* = 0.3 for the logit distillation loss, and *γ* = 0.1 for the feature-level loss. Together, these losses encourage the student not only to match the teacher’s output decisions but also to absorb its internal reasoning patterns, striking a balance between learning independently and imitating a stronger model.

### 3.9 Model II: ViT-KD with ConvNext-Tiny

Model II integrates a pipeline of teacher–student–ensemble framework with ViTs for KD and a complementary ConvNeXt-based CNN classifier, followed by a weighted fusion ensemble.

The proposed method uses a teacher–student model, where the teacher is a pre-trained frozen model that provides soft targets and features and the student is a smaller model trained to match both teacher outputs and feature representations. The second independent classifier for ensemble is ConvNeXt-Tiny, which is a modern CNN. This CNN picks texture differences that catch the subtle color changes. The ensemble is an independent model that is a fusion of predictions from models, ViT student with ConvNeXt.

The model employs a multi-objective KD strategy. The first component is logit distillation, in which the student model trains from the teacher’s softened output probabilities using KL divergence. This helps the student understand the correct class with the teacher’s confidence distribution across all classes. The second component is feature-level distillation, where the student is encouraged to match the teacher’s internal CLS token representation using MSE. Together, these two complementary mechanisms form a combined KD process designed to help the smaller student model to inherit both the predictive behavior and the internal representational structure of the larger teacher network. The model parameters are given in **Table 2**.

To guide the student model toward both accurate predictions and deeper understanding, the training process involves the three losses as described in models I and II. As always, the cross-entropy loss supervises the correct class identification. In addition, KL divergence-based distillation guides the student model to match the teacher’s soft probability distributions, generated using a temperature of 4, while appropriate scaling ensures stable gradient propagation during optimization. Complementing these two losses, a feature matching the MSE loss encourages the student to align its internal CLS token features with those of the teacher. As the student’s feature dimension is smaller, a projection layer is used before comparison. This extra feature-level supervision enables the student to absorb the teacher’s final decisions along with semantic representations.

The combination of these losses is done with percentage of importance, i.e., 60% importance to cross-entropy, 30% to KL divergence loss, and 10% to feature MSE loss, enabling the student to learn both the teacher’s final predictions and the reasoning behind them.

To train the networks, we used AdamW, a stable and widely used optimizer for transformer-based architectures. The student network is trained with a learning rate of 3*e*−4, whereas the ConvNeXt of the ensemble model uses a slightly lower learning rate of 2*e*−4, with a weight decay applied in both cases to reduce overfitting. Each model is trained for 30 epochs, selected based on the validation metrics, which are stored for use in the final ensemble. The model parameters of ConvNeXt-Tiny are shown in **Table 3**.

The proposed ensemble model achieved a notable 86.6% classification accuracy on the cashew dataset. The results demonstrated that, with the lightweight design of the student model, when paired with ensemble fusion, it can reach the capabilities of a much large transformer while remaining computationally efficient and suitable for real-world deployment. The 86.6% accuracy achieved indicates strong robustness in distinguishing unripe, ripe, and overripe cashew apples under challenging variations of lighting, texture, and natural appearance.

### 3.10 Model III: ViT-KD with EdgeNeXt-Small

**Figure 3:**
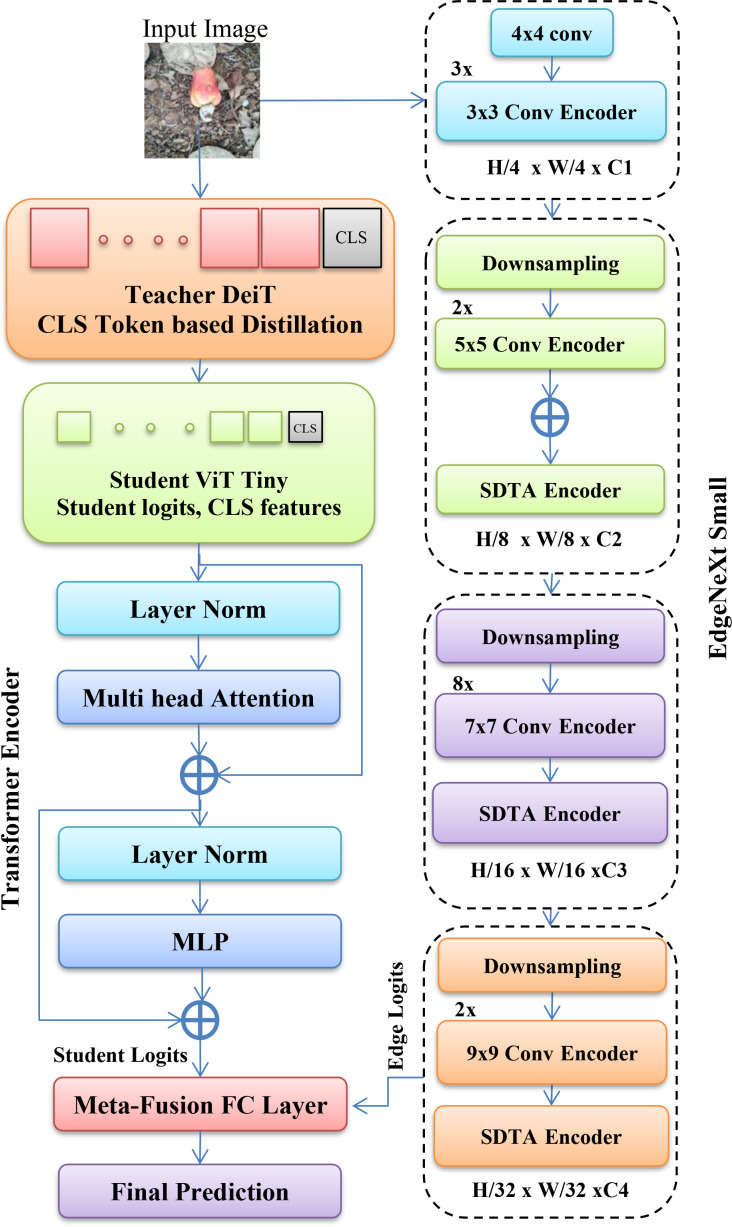
Proposed method architecture. Vision transformer–knowledge distillation (ViT-KD) with EdgeNeXt-Small.

Model III is the proposed framework that employs a multi-model architecture consisting of a frozen DeiT-Base teacher, a lightweight DeiT-Tiny student enhanced through KD, and an EdgeNeXt-Small convolutional network. The proposed architecture is shown in **Figure 3**. The teacher model, comprising approximately 86 million parameters, provides soft supervision and high-level semantic features. The student model, with nearly 6.3 million trainable parameters including a feature projection head, learns compact global representatives.

During KD, the student network is optimized using a weighted combination of hard-label cross-entropy loss (weight *α* = 0.6), KL divergence-based logit distillation (weight *β* = 0.3), temperature (*T* = 4.0), and MSE-based FD (weight *γ* = 0.1).

The proposed frameworks are trained in three stages: the knowledge-distilled student network optimized for 30 epochs, the EdgeNeXt-Small branch trained independently for 30 epochs, and a lightweight meta-fusion classifier trained for 10 epochs using frozen predictions from both networks. The optimizer AdamW with a learning rate of 2 × 10^−4^ and a weight decay of 1 × 10^−4^ was used. The model parameters are presented in **Table 5**.

The trained student model produces a class probability vector through the softmax operation representing the global semantic reasoning learned via KD. Similarly, EdgeNeXt-Small also generates its own probability vector, which encodes fine-grained local texture and edge information extracted through Conv-Attention blocks. These two probability vectors are concatenated to form a six-dimensional feature representation, which is fed to a lightweight meta-classifier, a linear or multinomial logistic regression layer to produce the final three-class label prediction.

**Meta-fusion strategy**

The averaging of the outputs from the two models was calculated as below, which is an alternative to the probability concatenation. The final prediction is computed using the weighted fusion and meta-classifier formulations described in Equations (7–10).

(7) $$P_{\text{final}}=wP_{\text{ViT}}+(1-w)P_{\text{Edge}}$$

Here, *w* is a fixed weight.

The meta-classifier learns adaptive fusion weights through the following trainable mapping:

(8) $$y\;=\;\sigma (W\cdot [P_{\text{ViT}},\;P_{\text{Edge}}]+\;b)$$

Here, $\left[P_{\text{ViT}},\;P_{\text{Edge}}\right]$ represents the concatenated probability vectors. Parameters *W* and *b* are learnable parameters. This formulation enables class-dependent and sample-specific weighting, allowing the fusion mechanism to dynamically emphasize the global semantic reasoning of ViT.

The meta-fusion mechanism therefore captures cross-model interactions and complementary strengths rather than relying on uniform blending. This enhances the discriminative capability of the fine-grained maturity classification where close visual differences separate different classes.

The overall KD-ViT with EdgeNeXt meta-ensemble workflow for three-class classification is summarized in **Algorithm 1**.

Algorithm

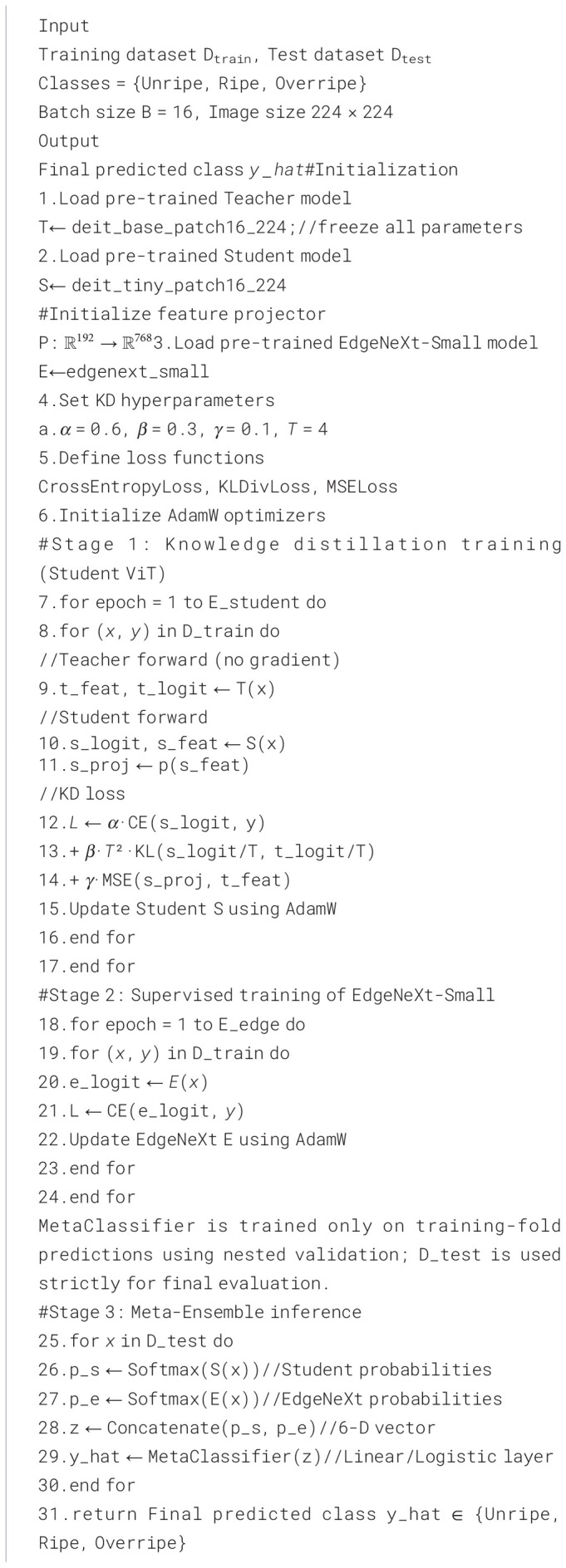

Overall, this model allows a light, efficient student transformer to learn from a heavier expert model, resulting in fast inference while retaining much of the teacher’s accuracy.

## 4 Results and discussion

In this study, we explored three KD frameworks designed to boost the performance of the lightweight student ViT. Each framework uses a powerful DeiT-Base teacher guiding a smaller DeiT-Tiny student. While the teacher remains frozen, it provides both logits and deep semantic CLS token features to guide the student during training. The three models are distinguished by the level of architectural support and complementary learners introduced alongside the primary KD mechanism.

**Table 6** presents the architectural progression of the proposed framework, highlighting the incremental integration of auxiliary lightweight models while maintaining identical distillation settings, from a pure KD approach to increasingly rich hybrid architectures, showing how auxiliary learners and stronger CNN counterparts can significantly enhance the classification ability of the student ViT while still keeping the model efficient and deployable. The quantitative performance progressions corresponding to these structural variations are as follows: i) ViT-KD + MobileNetV3, with accuracy of 83% and macro-F1 of 0.83; ii) ViT-KD + ConvNeXt, with accuracy of 86.6% and macro-F1 of 0.86; and iii) ViT-KD + EdgeNeXt, with accuracy of 90% and macro-F1 of 0.90. Comparative analysis of multiple deep learning architectures, including EfficientNet, ResNet50, DenseNet, ViT-KD combined with MobileNetV3, the hybrid ViT-Tiny (KD from DeiT-Base) with ConvNeXt, and ViT-Tiny (KD from DeiT-Base) with EdgeNeXt, highlighted significant differences in their ability to perform on fine-grained fruit maturity classification. Comparative experiments with conventional CNN baselines such as EfficientNet, ResNet50, DenseNet, and the lightweight CNN baselines such as SheffleNetV2 and MobileViT-S demonstrated that the proposed ViT-KD with EdgeNeXt ensemble achieved improved classification accuracy.

**Table 6:** Comparison of the three models.

Feature	ViT-KD with Mobile Net V3	ViT-KD with ConvNeXt	ViT-KD with EdgeNeXt-Small
Teacher	DeiT-Base	DeiT-Base	DeiT-Base
Student	DeiT-Tiny	DeiT-Tiny	DeiT-Tiny
Extra model	MobileNetV3-Small	ConvNeXt-Tiny	ConvNeXt-Tiny
KD loss	Weighted CE/KL/MSE	Weighted CE/KL/MSE	Weighted CE/KL/MSE
Loss weights	0.6/0.3/0.1	0.6/0.3/0.1	0.6/0.3/0.1
Feature alignment	Yes	Yes	Yes
Epochs	30 + 25	30 + 30	30 + 30
Ensemble	ViT with MobileNet V3 and Softmax	ViT with ConvNeXt-Tiny with Softmax	ViT with EdgeNeXt-Small with Meta Fusion

*ViT*, vision transformer; *KD*, knowledge distillation; *DeiT*, data-efficient image transformer; *CE*, cross-entropy; *KL*, Kullback–Leibler; *MSE*, mean squared error.

To improve the reliability of the final predictions, the code performs an automatic search to determine the most effective way to combine the outputs of two independently trained models, the ViT-based student and the EdgeNeXt network. Instead of choosing arbitrary ensemble weights, the script systematically tests every combination of weights between 0.1 and 0.98, blending the probability outputs of both models and evaluating each mixture using the multi-class receiver operating characteristic–area under the curve (ROC-AUC) score.

The score indicates how effectively the ensemble model distinguishes among the three classes of the cashew apple maturity stages. For each weight pair, the probabilities are computed, blended, and evaluated for effectiveness, with the combination that yields the highest AUC selected for computing the final predictions.

### 4.1 Evaluation criteria

The evaluation criteria including the statistical measures of different metrics such as accuracy and the F1 score were calculated. Furthermore, statistical cross-validation was performed for the evaluation of the proposed model. Since it is a multi-class classification, the results are presented class-wise.

### 4.2 Confusion matrix

The confusion matrix of the three classes is as follows:

(9) $$\left[\begin{array}{ccc} {\text{TP}}_{\text{Unripe}} & {\text{FP}}_{\text{Unripe}\to \text{Ripe}} & {\text{FP}}_{\text{Unripe}\to \text{Overripe}}\\ {\text{FP}}_{\text{Ripe}\to \text{Unripe}} & {\text{TP}}_{\text{Ripe}} & {\text{FP}}_{\text{Ripe}\to \text{Overripe}}\\ {\text{FP}}_{\text{Overripe}\to \text{Unripe}} & {\text{FP}}_{\text{Overripe}\to \text{Ripe}} & {\text{TP}}_{\text{Overripe}} \end{array}\right]$$

Together, these evaluation metrics provide a comprehensive view of the strength and limitations of the model. Validation loss is a key measure for identifying the model’s ability to generalize unseen data. Training loss identifies how well the model learns from its own examples, while validation loss shows learning generalization. It helps to determine whether the model is learning true patterns.

A low validation loss matching the training loss indicates a good generalization. An increase in the validation along with decreasing training loss means that the model is overfitting. The model is underfitting if both losses increase. Tracking these loss patterns across the epochs reveals how stable and effective the learning process is.

**Figure 4:**
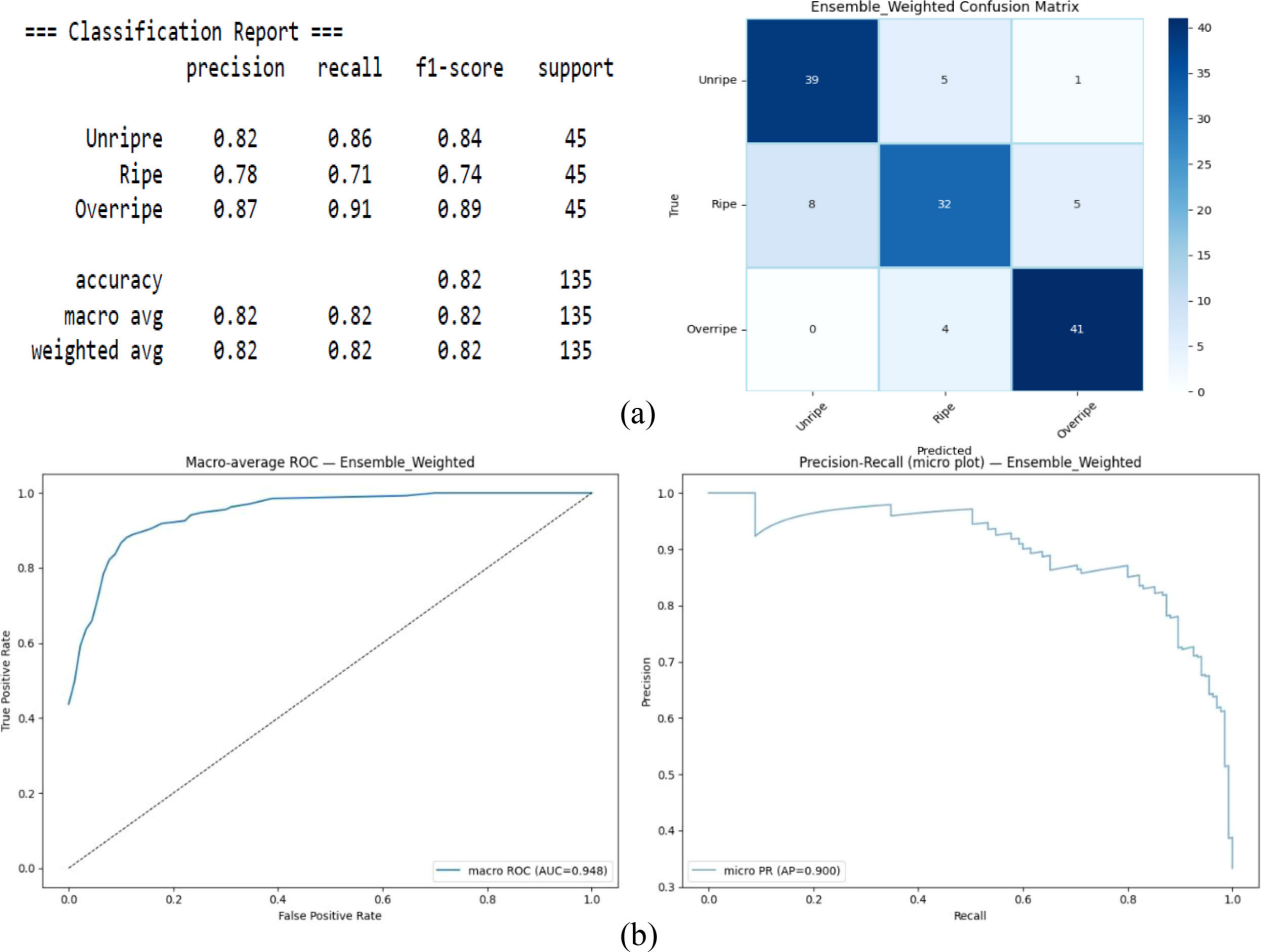
Vision transformer–knowledge distillation (ViT-KD) with Mobile Net-V3. **(A)** Confusion matrix. **(B)** Receiver operating characteristic (ROC) and precision–recall plots.

The formula for the calculation of the validation loss is as follows:

(10) $$\text{Validation Loss}=\frac{1}{N}\sum_{i=1}^{N}L(y_{i},\;{\hat{y}}_{i})$$

where *N* is the number of validation samples, $y_{i}$ is a true label, ${\hat{y}}_{i}$ is the model prediction, and L is the loss function. **Figure 4** presents the classification performance of the weighted ensemble model. The overall macro-F1 score (0.82) and the AUC (0.948) indicate strong discriminative ability. However, the class-wise metrics revealed systematic patterns in misclassification. The Ripe class exhibited the lowest recall (0.71) compared with the Unripe (0.86) and Overripe (0.91) classes, indicating that a proportion of true Ripe samples were incorrectly predicted as adjacent classes. The Overripe class attained the highest recall of 0.91, indicating clearer visual characteristics at advanced maturity stages. Although the ROC curve (AUC = 0.948) reflects strong overall separability, some confusion remains between adjacent classes. The precision–recall curve [average precision (AP) = 0.900] further showed that precision decreases as recall approaches 1.0, implying that maximizing recall increases false positives, mainly between the unripe and ripe samples.

In contrast, Overripe achieved the highest recall (0.91), suggesting that late-stage maturity possesses more visually distinct characteristics. The ROC curve (AUC = 0.948) confirmed the strong separability overall; however, a high AUC does not eliminate boundary-level confusion between visually similar neighboring classes. Similarly, the precision–recall curve (AP = 0.900) demonstrated stable precision at moderate recall levels, but showed a decline as recall approaches 1.0, indicating that forcing full recall introduces false positives primarily between Unripe and Ripe.

**Table 7** shows the key hyperparameters used to train the DeiT-Tiny student, ConvNeXy-Tiny, and EdgeNeXt-Small models. Each model uses 224 × 224 × 3 images and uniformly trained for 30 epochs with a batch size equal to 16. The optimization technique used was AdamW, which ensures stable training. The GELU activation function used supports smooth nonlinear transformations. The learning rate values ranged between 0.0003 and 0.0002, depending on the optimization sensitivity of each model.

**Table 7:** Hyperparameters.

Hyperparameters	Student (ViT-Tiny KD)	ConvNeXt-Tiny	EdgeNeXt
Epochs	30	30	30
Learning rate	3*e*−4	2*e*−4	2*e*−4
Batch size	16	16	16
Input size	224 × 224 × 3	224 × 224 × 3	224 × 224 × 3
Optimizer	AdamW	AdamW	AdamW
Activation function	GELU	GELU	GELU
Output function	Softmax	Softmax	Softmax

*ViT*, vision transformer; *KD*, knowledge distillation; *GELU*, Gaussian error linear unit.

**Figure 5:**
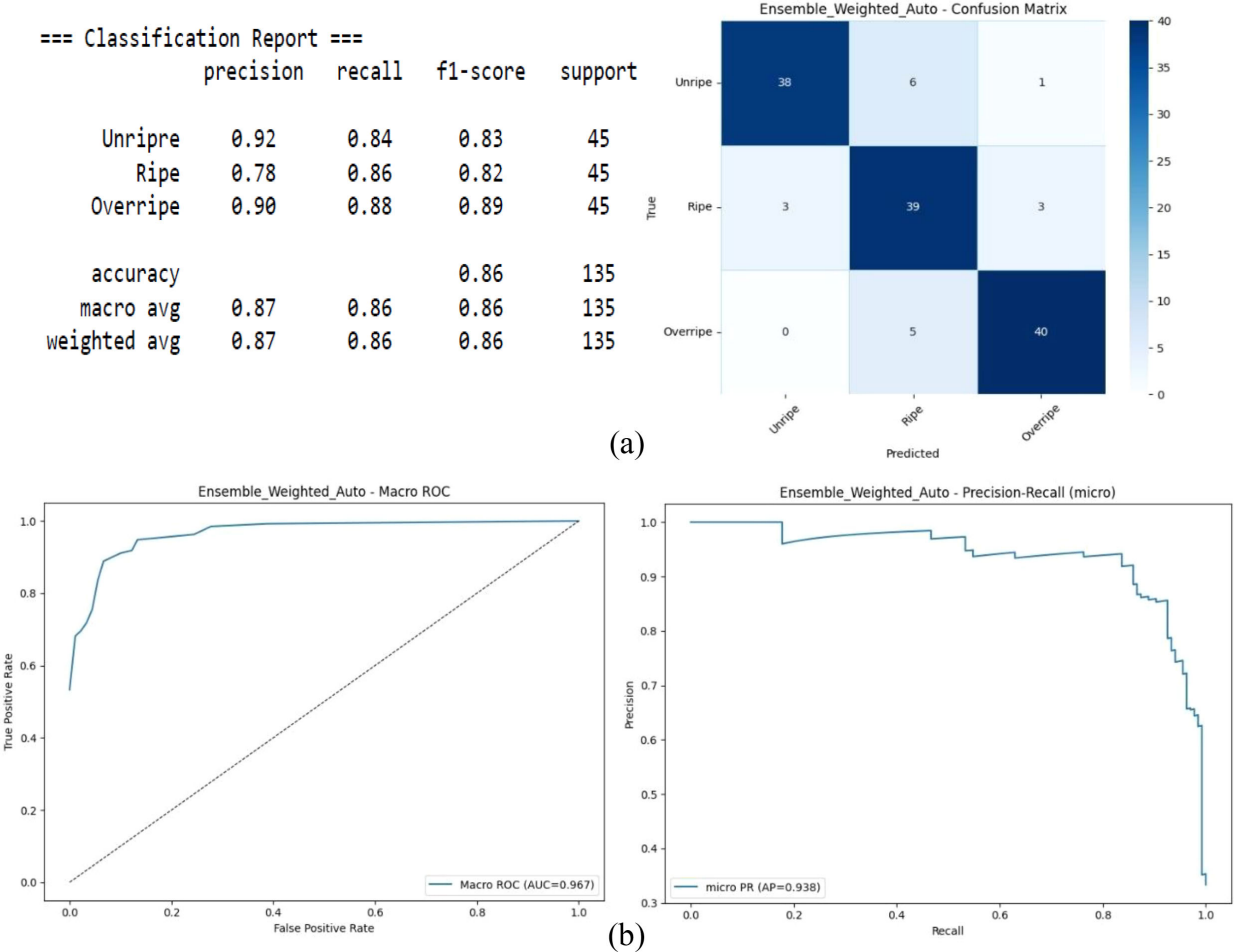
Vision transformer–knowledge distillation (ViT-KD) with ConvNeXt. **(A)** Confusion matrix. **(B)** Receiver operating characteristic (ROC) and precision–recall.

**Figure 5** illustrates the performance of the model. The overall accuracy increased to 86%, with a macro-average F1 score of 0.86, showing the better class balance compared with that of the previous model. The macro-ROC curve and AUC of 0.967 indicates the separability among the three classes. Similarly, the micro-average precision–recall of 0.938 shows stable precision for different recall values.

**Figure 6:**
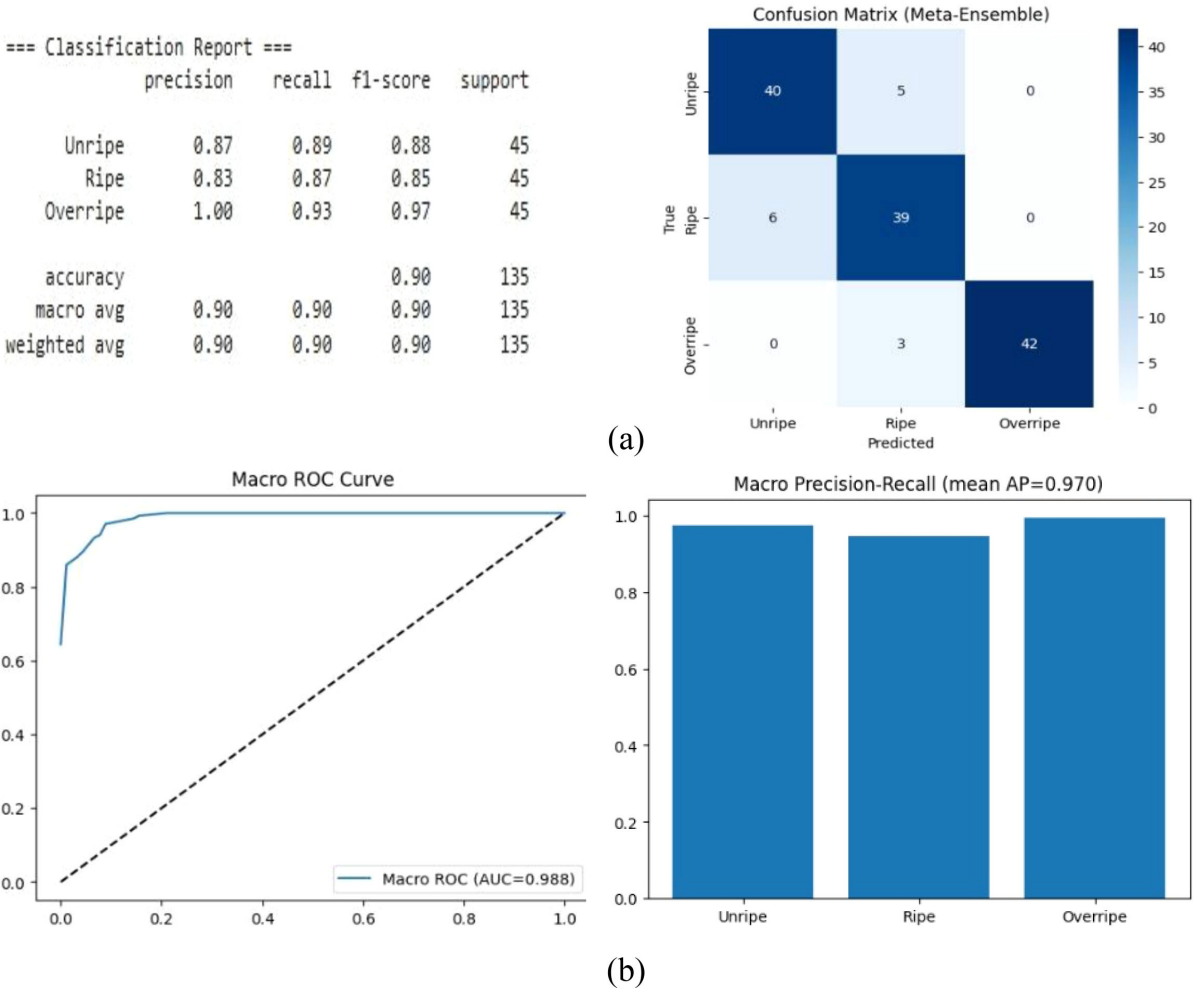
Vision transformer–knowledge distillation (ViT-KD) with EdgeNeXt-Small. **(A)** Confusion matrix. **(B)** Receiver operating characteristic (ROC) and precision–recall.

The model results are illustrated in **Figure 6**. During the initial epochs, both losses, i.e., training and validation, decreased steadily, indicating the model’s steady and effective learning of the visual patterns associated with cashew apple maturity. While the training progressed, the validation loss began to level off while the training loss continued declining, suggesting that the model reached an optimal learning point without overfitting.

The close alignment of the curves in the later epochs demonstrated that the student DeiT-Tiny and EdgeNeXt models retained strong generalization capabilities even after KD. The stable validation performance suggests that the ensemble model captured reliable and transferable features for classifying unripe, ripe, and overripe cashew apples.

The results of the ensemble ViT-KD with EdgeNeXt, given in **Figure 6**, showed 90% accuracy with a balanced macro, precision, recall, and F1 scores. The fivefold cross-validation yielded a mean accuracy of 86.89% ± 2.89%, which is an indication of stable performance across partitions. The ROC curve with AUC = 0.988 shows good separability. The precision–recall analysis with a mean AP = 0.970 demonstrates strong confidence in predictions across all classes.

Despite the strong overall performance, a small number of misclassifications still occurred, mainly between the Unripe and Ripe categories. Both classes achieved high recall: 0.89 for Unripe and 0.87 for Ripe. However, for the gradual transition of color from the early-stage ripe fruits to the late-stage unripe fruits, minor confusion exists. In comparison, the Overripe class was classified with high reliability (F1 = 0.97, precision = 1.00), indicating that the later maturity stages possess clearer visual characteristics.

**Figure 7:**
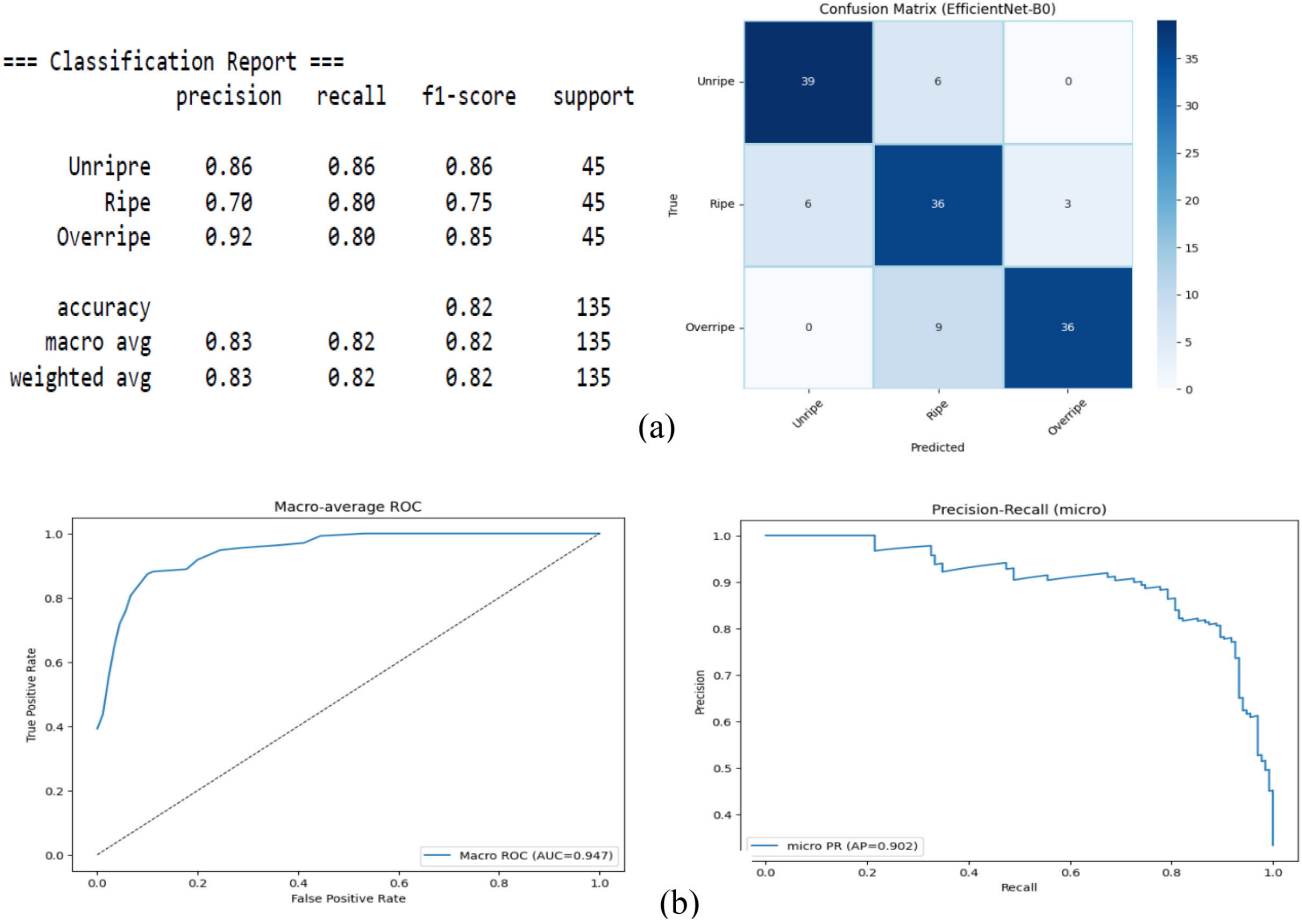
EfficientNet. **(A)** Confusion matrix. **(B)** Receiver operating characteristic (ROC) and precision–recall.

The results of EfficientNet are shown in **Figure 7**, which showed an overall accuracy of 82% and a macro-F1 score of 0.82. Despite the ROC curve with AUC = 0.947 indicating good overall separability, the class-wise analysis showed an imbalance in performance. The lowest precision of 0.70 and an F1 score of 0.75 indicate false positives or boundary cases in the Ripe class. The Unripe class maintained stable performance, with performance F1 = 0.86, while Overripe showed high precision of 0.92.

**Figure 8:**
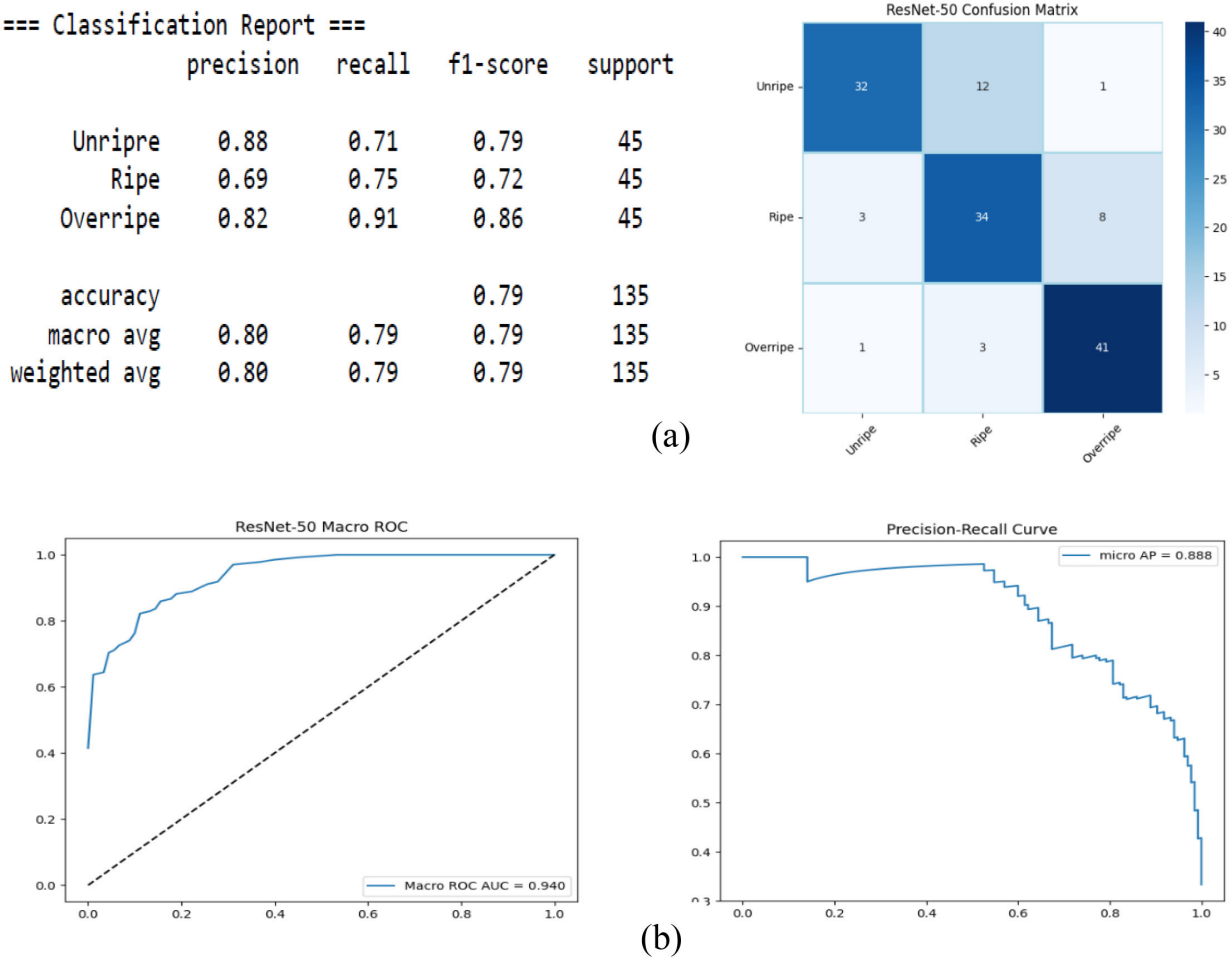
ResNet-50. **(A)** Confusion matrix. **(B)** Receiver operating characteristic (ROC) and precision–recall.

**Figure 8** presents the performance of the ResNet-50 baseline model, which achieved an overall accuracy of 79% and a macro-F1 score of 0.79. While the ROC curve (AUC = 0.940) indicates reasonable global separability, the class-wise metrics revealed notable weaknesses in intermediate maturity detection. In particular, the Ripe class recorded the lowest precision (0.69) and F1 score (0.72), suggesting that the model frequently confuses the ripe samples with the adjacent maturity stages.

**Figure 9:**
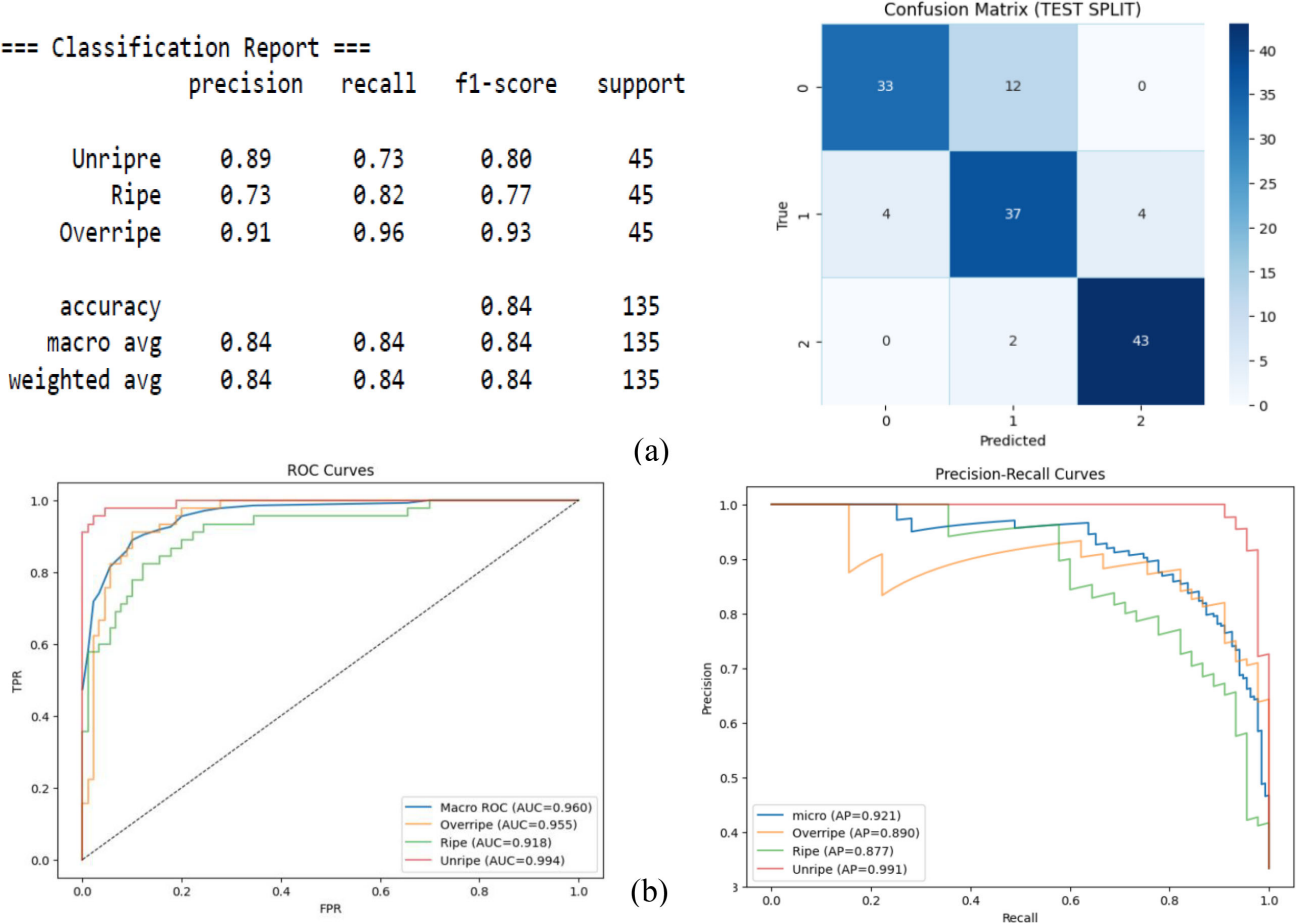
DenseNet. **(A)** Confusion matrix. **(B)** Receiver operating characteristic (ROC) and precision–recall.

**Figure 9** shows an overall accuracy of 84%, with macro-precision, recall, and F1 scores all around 0.84, indicating a balanced class performance. The ROC curves with macro-AUC = 0.960 demonstrate strong class separability, and the class-wise AUC suggests that the Unripe class was distinguished well, with an AUC = 0.994. The precision–recall showed high average precision: AP = 0.991 for the Unripe class and AP = 0.877 for the Ripe class. This decline in precision suggests the difficulty faced by the model in the transitional maturity stages.

The knowledge transfer between teacher and student allowed the hybrid ensemble models to form more robust class boundaries and generalize better across variations in illumination, size, and fruit orientation compared with models trained without distillation. The results confirmed that fruit maturity grading characterized by subtle intra-class differences and high inter-class similarity benefits from a modeling approach that jointly incorporates local texture extraction, global context reasoning, and distilled high-level semantic knowledge. The detailed performance comparison of the proposed models with the others are presented in **Table 8**. Also the comparison between the accuracy measure alone presented in **Table 9**.

**Table 8:** Comparison of the precision, F1 score, and recall of the models.

Model	Precision			F1 score			Recall		
	Ripe	Unripe	Overripe	Ripe	Unripe	Overripe	Ripe	Unripe	Overripe
EfficientNet	0.7	0.86	0.92	0.75	0.86	0.85	0.8	0.86	0.8
ResNet50	0.69	0.88	0.82	0.72	0.79	0.86	0.75	0.71	0.91
DenseNet	0.73	0.91	0.89	0.77	0.93	0.8	0.82	0.96	0.73
ViT-KD with MobileNet V3	0.78	0.82	0.87	0.74	0.84	0.89	0.71	0.86	0.91
ViT-Tiny (KD from DeiT-Base) with ConvNeXt	0.8	0.83	0.95	0.79	0.84	0.91	0.8	0.88	0.88
ViT-Tiny (KD from DeiT-Base) with EdgeNeXt	0.83	0.87	1.00	0.85	0.88	0.97	0.87	0.89	0.93

*ViT*, vision transformer; *KD*, knowledge distillation.

**Table 9:** Comparison of the accuracy of the models.

Model	Accuracy(%)
ResNet50	79
MobileViT-S	81
EfficientNet	82
DenseNet	84
ViT-KD with MobileNet V3	83
ViT-Tiny with ConvNeXt	86.6
ShuffleNetV2	86.6
ViT-KD with EdgeNeXt	90

*ViT*, vision transformer; *KD*, knowledge distillation.

In order to attain statistical robustness and address the concerns related to a small dataset size, a fivefold cross-validation was performed to evaluate the performance stability across multiple data partitions. The model achieved accuracy of 86.89% ± 2.89%, an F1 score of 0.8689 ± 2.88, and a mean recall of 86.89 ± 2.89. The 2% standard deviation relatively across all phases indicates consistent performance and limited sensitivity, which corresponds to the internal robustness within the data constraints. There is no evidence of data leakage as the validation or the test phase did not reuse the test results for the final evaluation.

The stand-alone distilled ViT-Tiny(KD) model comprises approximately 5.278 million parameters, with a model size of 20.30 MB and a computational complexity of 0.960 GFLOPs. It achieved an average inference latency of 8.79 ms per image, making it suitable for real-time deployment in edge environments.

The stand-alone distilled ViT-Tiny achieved 83.33% accuracy prior to fusion. However, the ensemble ViT-Tiny with EdgeNeXt achieved the best performance of 90% on held-out split and 86.89% ± 2.89% under fivefold cross-validation. An overall 6.7% improvement was shown by the ensemble model, and at no stage were the validation or the test samples reused for the final evaluation, preventing data leakage. While the ensemble introduces additional computational overhead, the stand-alone student remains highly efficient (8.79 ms latency), allowing deployment flexibility depending on the performance *versus* efficiency requirements.

Many misclassifications occurred between the unripe and ripe sample images. Visual inspection of representative failure cases showed that transitional color stages and illumination variations reduced the contrast between these adjacent classes. As maturation is continuous while labeling is discrete, minor boundary ambiguity is expected.

Although the dataset is limited to only 900 images, different mechanisms were integrated in the proposed model by considering the field conditions. Augmentation ensures that the images captured at the fields involve various positions, and this step ensures that such variety of images will be used during the training process. The next step involved in the proposed model is KD, which handles the soft probability distributions compared with the hard labels. The DeiT-Base teacher-to-student model, which is a lightweight setup, proposed in this work handles the inter-class relationships, which are too similar. The next-level architecture transfers the soft probability distributions, and additional feature-level alignment of the CLS tokens further enables the student to inherit the teacher’s internal semantic representations. Thirdly, the hybrid integration of EdgeNeXt-Small introduces stronger convolutional feature extraction.

## 5 Conclusion

Cashew apple maturity grading remains a relatively underexplored problem largely due to the limited availability of well-curated datasets. In this study, we evaluated several state-of-the-art deep learning architectures, including conventional CNN models such as EfficientNet, ResNet50, and DenseNet, as well as lightweight networks such as MobileViT-S and ShuffleNetV2, along with ViT-based KD frameworks, to classify cashew apples into three maturity stages: Unripe, Ripe, and Overripe. The experimental results showed that, although the traditional CNN models performed well in general object recognition tasks, they struggled to capture the subtle visual differences required for fine-grained maturity classification. In contrast, the proposed ensemble framework with a DeiT-Base teacher, a distilled DeiT-Tiny student, and an auxiliary EdgeNeXt model demonstrated superior performance. The model achieved 90% accuracy on a held-out split, while fivefold cross-validation produced a mean accuracy of 86.89% ± 2.89%, showing stable performance across different data partitions. Moreover, the lightweight model enables efficient inference, making it well suited for potential deployment in edge-based agricultural applications.

## Data Availability

Publicly available datasets were analyzed in this study. This data can be found here: https://ieee-dataport.org/documents/goa-cashew-apple-maturity-grading.
